# Targeting ESM-1 inhibits spinal metastasis by disrupting tumor-endothelial adhesion

**DOI:** 10.1186/s43556-025-00332-0

**Published:** 2025-10-30

**Authors:** Seong Jun Kim, Wan-Kyu Ko, Daye Lee, Min Je Kim, Gi-Beom Ju, Zixiang Luo, Je-Beom Hong, Seung Hun Sheen, Seil Sohn

**Affiliations:** 1 Department of Neurosurgery, CHA Bundang Medical Center, CHA University College of Medicine, 59, Yatap-Ro, Bundang-Gu, Seongnam-si, Gyeonggi-do 13496 Republic of Korea; 2 Department of Biomedical Science, CHA University, 335, Pangyo-Ro, Bundang-Gu, Seongnam-Si, Gyeonggi-Do 13488 Republic of Korea; 3 Department of Chemistry and Chemical Biology, Rutgers, The State University of New Jersey, 123 Bevier Road, Piscataway, NJ 08854 USA; 4 Department of Spine Surgery and Orthopaedics, Xiangya Hospital, Central South University, Changsha, 410008 China; 5 Department of Neurosurgery, Kangbuk Samsung Hospital, Sungkyunkwan University School of Medicine, Seoul, 03181 Republic of Korea

Dear Editor,

Spinal metastasis is a major cause of cancer-associated morbidity, yet effective therapeutic strategies remain limited and current management primarily focuses on symptom control [1]. Endothelial cell-specific molecule 1 (ESM-1) has been implicated in the metastatic progression of various cancers, including melanoma, breast cancer, and lung cancer, where its overexpression correlates with a poor prognosis. Additionally, studies involving ESM-1 knockout models have demonstrated reduced leukocyte transmigration and vascular permeability, suggesting its involvement in tumor-endothelial interactions and metastatic dissemination [2]. Nevertheless, the precise mechanisms by which ESM-1 contributes to spinal metastasis remain poorly characterized. In this study, we hypothesized that ESM-1 silencing could interfere with tumor-endothelial interactions, thereby reducing spinal metastasis by disrupting key adhesion pathways within the metastatic cascade.

To investigate the role of ESM-1 in spinal metastatic breast cancer, we generated a spinal metastatic breast cancer cell line following the protocol of Xiao et al. [3]. Human breast cancer MDA-MB-231 cells expressing firefly luciferase and puromycin resistance (P0-MDA) were inoculated into immunodeficient mice via an intracardiac injection. Tumor cells that had metastasized to the spine were isolated, selected with puromycin, and expanded in culture to generate the P1-MDA cell line. To further enhance the spinal metastatic potential, P1-MDA cells were re-inoculated, and additional spinal metastatic cells were isolated and expanded as the P2-MDA cell line. Spinal metastases were monitored by bioluminescence imaging (BLI; Fig. 1a, top) and confirmed a significant increase in both ESM-1 mRNA and protein levels in the isolated tumors as the cells progressed from P0-MDA to P1-MDA and P2-MDA (Fig. 1a, bottom). Collectively, these findings demonstrate a progressive upregulation of ESM-1 that parallels the acquisition of enhanced metastatic potential.

**Fig. 1:**
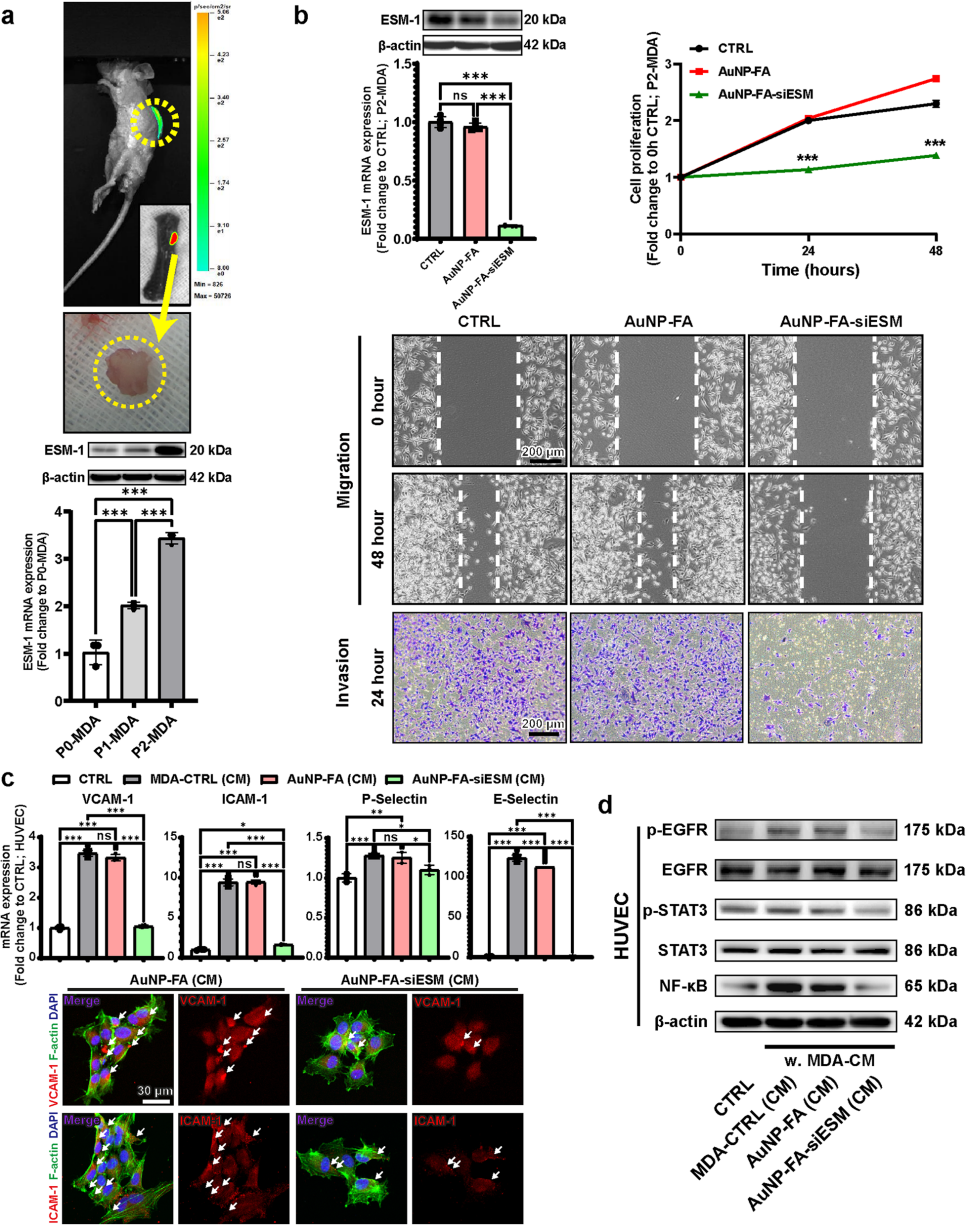
Knockdown of ESM-1 by AuNP-FA-siESM in spinal metastatic breast cancer cells suppresses the expressions of adhesion molecules in endothelial cells. **a** Representative bioluminescence images of spinal metastases in mice (top) and isolated metastatic tumors from the spine (middle). Validation of ESM-1 overexpression in P0-MDA, P1-MDA, and P2-MDA cells by Western blotting and qRT-PCR (bottom). **b** ESM-1 knockdown of AuNP-FA-siESM was confirmed by Western blotting and qRT-PCR (top, left). Quantification of cell proliferation at 0, 24, and 48 h (top, right). A wound healing assay was performed to examine the effect of ESM-1 knockdown on cell migration (middle). An invasion assay performed using Matrigel Transwell assays (bottom). **c** Human umbilical vein endothelial cells (HUVEC) were cultured with a conditioned medium (CM) from P2-MDA cells treated with AuNP-FA or AuNP-FA-siESM. Relative mRNA expression levels of vascular cell adhesion molecule-1 (VCAM-1), intercellular adhesion molecule-1 (ICAM-1), P-Selectin, and E-Selectin in HUVEC exposed to CM from MDA-CTRL-treated, AuNP-FA-treated, or AuNP-FA-siESM-treated P2-MDA cells, as quantified by qRT-PCR (top). Representative immunofluorescence images of VCAM-1 and ICAM-1 in HUVEC (bottom). White arrows indicate fluorescent signals corresponding to adhesion molecule expression. **d** Western blot analysis of p-EGFR, EGFR, p-STAT3, STAT3, NF-κB, and β-actin in HUVEC treated with CM from MDA-CTRL-treated, AuNP-FA-treated, or AuNP-FA-siESM-treated P2-MDA cells. Results were analyzed by a one-way ANOVA with Tukey’s post hoc test (ns, not significant; ^*^p < 0.05; ^**^p < 0.01; ^**^.^*^p < 0.001)

To evaluate the effect of ESM-1 knockdown in spinal metastatic breast cancer, we synthesized a siRNA delivery system utilizing gold nanoparticles (AuNPs). The AuNPs were prepared via direct reduction with poly-ethylenimine (PEI), followed by sequential electrostatic conjugation of folate-modified polyethylene glycol (FA-PEG) and ESM-1 siRNA (siESM) onto the AuNP surface (Fig. S1a). The FA-conjugated AuNPs loaded with siESM (AuNP-FA-siESM) were characterized using transmission electron microscopy (Fig. S1b, left), a zeta potential analysis (Fig. S1b, right), and Fourier-transform infrared spectroscopy (Fig. S1c, left), confirming successful incorporation. The cumulative siRNA release from AuNP-FA-siESM was examined over 96 h using a SYBR Gold assay (Fig. S1c, right). At 1 h, 13% of siRNA was released, which increased to 63% at 24 h and reached complete release by 96 h.

The FA-functionalization was designed to enhance the selective internalization of the siRNA delivery system by metastatic breast cancer cells, which frequently overexpress folate receptors. As shown in Fig. S1d, P2-MDA cells treated with AuNP-FA-siESM exhibited significantly enhanced intracellular uptake compared to those treated with AuNP-PEG-siESM. Taken together, these findings confirm the successful synthesis of AuNP-FA-siESM and demonstrate that FA functionalization enhances tumor-targeting efficiency. However, a limitation of this experiment is that the tumor-targeting efficiency and siRNA release profile were characterized exclusively in an in vitro model. Future studies are needed to evaluate the efficacy and biodistribution of the AuNP-FA-siESM system in an in vivo model.

To investigate the knockdown efficiency of the AuNP-FA-siESM treatment in spinal metastatic breast cancer cells, P2-MDA cells were treated with AuNP-FA-siESM. The ESM-1 mRNA expression and protein levels showed no significant differences between the untreated control (CTRL) and AuNP-FA groups, whereas the AuNP-FA-siESM group exhibited a significant reduction in ESM-1 mRNA and protein levels compared to the CTRL group (Fig. 1b, top-left). Cell proliferation rates showed no significant differences between the CTRL group and AuNP-FA group at any time point (Fig. 1b, top-right). However, cell proliferation in the AuNP-FA-siESM group was significantly reduced compared to that in the CTRL group at 24 and 48 h. Furthermore, wound healing and invasion assays demonstrated that AuNP-FA-siESM treatment significantly inhibited cancer cell migration and invasion, respectively (Fig. 1b, middle and bottom). Collectively, these results indicate that AuNP-FA-siESM effectively suppresses ESM-1 expression in spinal metastatic breast cancer cells, leading to reduced cell proliferation, migration, and invasion, thereby inhibiting cancer progression.

In the metastatic cascade, cancer cell adhesion to the endothelial cells of target organs is a critical step [4]. To assess the impact of ESM-1 expression on this process, HUVEC were treated with a conditioned medium (CM) from P2-MDA cells in which ESM-1 was suppressed and the expression levels of key adhesion molecules were analyzed. A quantitative mRNA analysis showed that vascular cell adhesion molecule-1 (VCAM-1) expression was significantly elevated in HUVEC following the treatment with CM from P2-MDA cells (MDA-CTRL (CM)) compared with untreated HUVEC (Fig. 1c, top). Notably, CM from P2-MDA cells treated with vehicle (AuNP-FA (CM)) did not reduce this increase. However, CM from P2-MDA cells with suppressed ESM-1 expression via AuNP-FA-siESM (AuNP-FA-siESM (CM)) significantly reduced VCAM-1 expression in HUVEC compared to the CTRL group. A similar pattern was observed for other adhesion molecules, including intercellular adhesion molecule-1 (ICAM-1), P-Selectin, and E-Selectin.

To validate these findings further, immunocytochemistry analyses were conducted for VCAM-1 and ICAM-1 in endothelial cells. Consistent with the mRNA results, HUVEC treated with CM from vehicle-treated P2-MDA cells exhibited elevated VCAM-1 and ICAM-1 expression levels, whereas CM from P2-MDA cells with suppressed ESM-1 expression via AuNP-FA-siESM markedly reduced the expression of both markers compared to the AuNP-FA group (Fig. 1c, bottom). Collectively, these data indicate that ESM-1 suppression in spinal metastatic cancer cells modulates the expression of adhesion molecules in endothelial cells, potentially critical for metastasis.

EGFR signaling is implicated in metastatic dissemination and adaptation to the tumor microenvironment [5]. To examine the role of ESM-1 in metastatic signaling and its impact on tumor-endothelial interactions, we investigated whether tumor-derived ESM-1 influences EGFR-related signaling in endothelial cells (Fig. 1d).

MDA-CTRL (CM) significantly increased p-EGFR levels in HUVEC compared to CTRL. Similarly, AuNP-FA (CM), in which ESM-1 was not silenced, also induced a significant increase in p-EGFR levels, indicating that ESM-1-expressing spinal metastatic breast cancer cells promote EGFR activation in endothelial cells. In contrast, AuNP-FA-siESM (CM) significantly reduced p-EGFR levels in HUVEC, suggesting that ESM-1 suppression in spinal metastatic breast cancer cells attenuates EGFR activation in endothelial cells. Further analysis of inflammatory signaling in endothelial cells revealed that p-STAT3 and NF-κB, key regulators of endothelial activation and vascular remodeling, were significantly upregulated in HUVEC treated with CM from MDA-CTRL and AuNP-FA (CM). However, AuNP-FA-siESM (CM) significantly reduced p-STAT3 and NF-κB levels in HUVEC. Collectively, these results suggest that tumor-derived ESM-1 promotes endothelial activation by inducing pro-inflammatory signaling cascades and contributes to tumor-endothelial interactions by modulating EGFR/NF-κB signaling.

In summary, this study demonstrates that ESM-1 plays a crucial role in spinal metastatic breast cancer progression and that its suppression via AuNP-FA-siESM effectively reduces metastatic potential by disrupting tumor-endothelial interactions. Although these findings are based on in vitro models, this study represents, to the best of our knowledge, the first report identifying ESM-1 as a potential therapeutic target for spinal metastatic breast cancer cells. Our findings suggest that ESM-1 contributes to both tumor-intrinsic signaling and the metastatic microenvironment, providing a strong rationale for future studies to validate its therapeutic potential in vivo and to investigate its relevance in other types of cancer.

## Supplementary Information

Supplementary Material 1.

## Data Availability

The data used to support the findings of this study are included within the article.

## References

[1] Kassamali RH, Ganeshan A, Hoey ETD, Crowe PM, Douis H, Henderson J. Pain management in spinal metastases: the role of percutaneous vertebral augmentation. Ann Oncol. 2011;22(4):782–6. 10.1093/annonc/mdq605. 20966180 10.1093/annonc/mdq605

[2] Rocha SF, Schiller M, Jing D, Li H, Butz S, Vestweber D, et al. Esm1 modulates endothelial tip cell behavior and vascular permeability by enhancing VEGF bioavailability. Circ Res. 2014;115(6):581–90. 10.1161/CIRCRESAHA.115.304718. 25057127 10.1161/CIRCRESAHA.115.304718

[3] Cai X, Luo J, Yang X, Deng H, Zhang J, Li S, et al. In vivo selection for spine-derived highly metastatic lung cancer cells is associated with increased migration, inflammation and decreased adhesion. Oncotarget. 2015;6(26):22905–17. 10.18632/oncotarget.4416. 26090868 10.18632/oncotarget.4416 PMC4673208

[4] Ellenrieder V, Adler G, Gress TM. Invasion and metastasis in pancreatic cancer. Ann Oncol. 1999;10(Suppl 4):46–50. 10.1093/annonc/10.suppl_4.S46. 10436784

[5] Nguyen DX, Bos PD, Massague J. Metastasis: from dissemination to organ-specific colonization. Nat Rev Cancer. 2009;9(4):274–84. 10.1038/nrc2622. 19308067 10.1038/nrc2622

