# Supplementary material for: Targeting ESM-1 inhibits spinal metastasis by disrupting tumor-endothelial adhesion
Source: Mol Biomed. 2025 Oct 30;6:90. doi: 10.1186/s43556-025-00332-0 (PMC12572558; doi:10.1186/s43556-025-00332-0)
Supplement: Supplementary file 1 — Supplementary Material 1. [file 43556_2025_332_MOESM1_ESM.docx]

**Supplementary Materials for**

Targeting ESM-1 Inhibits Spinal Metastasis by Disrupting Tumor-Endothelial Adhesion

Seong Jun Kim ^1,2^, Wan-Kyu Ko ^3^, Daye Lee ^1,2^, Min Je Kim ^1,2^, Gi-Beom Ju ^1,2^, Zixiang Luo ^4^, Je-Beom Hong ^5^, Seung Hun Sheen ^1^, and Seil Sohn ^1,2*^

^1^ Department of Neurosurgery, CHA Bundang Medical Center, CHA University; 59, Yatap-ro, Bundang-gu, Seongnam-si, Gyeonggi-do, 13496, Republic of Korea

^2^ Department of Biomedical Science, CHA University; 335, Pangyo-ro, Bundang-gu, Seongnam-si, Gyeonggi-do, 13488, Republic of Korea

^3^ Department of Chemistry and Chemical Biology, Rutgers, The State University of New Jersey, 123 Bevier Road, Piscataway, NJ, 08854, USA

^4^ Department of Spine Surgery and Orthopaedics, Xiangya Hospital, Central South University, Changsha, 410008, China

^5^ Department of Neurosurgery, Kangbuk Samsung Hospital, Sungkyunkwan University School of Medicine, Seoul, 03181, Republic of Korea

^*^Seil Sohn, MD, PhD

Department of Neurosurgery,

CHA University College of Medicine

59, Yatap-ro, Bundang-gu, Seongnam-si, Gyeonggi-do, 13496, Korea

Phone: 82-31-881-7966

Fax: 82-2-780-5269

E-mail: sisohn@cha.ac.kr

^*^ Seil Sohn is the corresponding author.

**Materials and Methods**

**Cell culture**

P0-MDA cells (expressing firefly luciferase and puromycin resistance; GenTarget Inc., San Diego, USA) were cultured in Dulbecco’s Modified Eagle’s Medium (DMEM; Invitrogen) supplemented with 10% fetal bovine serum (FBS) and 1% penicillin–streptomycin at 37 ℃ in a 5% CO_2_ atmosphere. Human umbilical vein endothelial cells (HUVEC; CEFO Bio, Seoul, Korea) were cultured in CEFOgro^™^ HUVEC Growth Medium containing 0.5% penicillin–streptomycin under identical conditions.

**Generation of spinal metastatic breast cancer cells**

The protocol for isolating tumor cells from spinal metastatic lesions was adapted from Xiao et al. [1]. A spinal metastatic breast cancer model was established by injecting 1 × 10^5^ P0-MDA cells into the left ventricle of female BALB/c nu/nu mice (4-6 weeks old). Metastases were monitored weekly using bioluminescence imaging (BLI; DavinchQ, Seoul, Korea). To derive metastatic cell lines, BLI-positive vertebral tumors were harvested, minced, and digested with collagenase/hyaluronidase for 2 hours at 37 °C. The suspension was filtered, and tumor cells were selected with 1 μg/mL puromycin for one week to establish the P1-MDA cell line. Following expansion in culture, P1-MDA cells were reinjected into the left ventricle to enhance their spinal metastatic potential. Once spinal metastasis was detected, P2-MDA cells were isolated and expanded for further experiments.

**Quantitative real-time polymerase chain reaction (qRT-PCR)**

Total RNA was extracted using the TRIzol reagent (Thermo Scientific, IL, USA) and reverse-transcribed to cDNA using a Maxime RT PreMix Kit (iNtRON Biotechnology, Seoul, Korea). qRT-PCR was performed as described previously [2]. Relative gene expression levels were normalized to glyceraldehyde-3-phosphate dehydrogenase (GAPDH) using the 2^−ΔΔCT^ method. All primers were obtained from Bioneer (Daejeon, Korea), with sequences provided in **Supplementary Table S1**.

**Western blotting**

Proteins were extracted using RIPA buffer (Thermo Scientific) with added protease and phosphatase inhibitors, and quantified with a BCA assay. Equal protein amounts were separated via SDS-PAGE and transferred to PVDF membranes (Millipore, MA, USA). Membranes were blocked with 5% skim milk for one hour and incubated overnight with primary antibodies: anti-ESM-1 (1:1,000; Abcam, Cambridge, UK), anti-phosphorylated epidermal growth factor receptor (p-EGFR; 1:1,000; Cell Signaling Technology, MA, USA), anti-EGFR (1:1,000; Cell Signaling Technology), anti-hypoxia inducible factor-1α (1:1,000; Cell Signaling Technology), anti-phosphorylated signal transducer and activator of transcription 3 (p-STAT3; 1:1,000; Cell Signaling Technology), anti-STAT3 (1:1,000; Cell Signaling Technology), anti-nuclear factor kappa-light-chain-enhancer of activated B cells (1:1,000; Cell Signaling Technology), and anti-β-actin (1:1,000; Applied Biological Materials Inc., Vancouver, Canada). Membranes were washed and incubated with HRP-conjugated secondary antibodies. Protein bands were detected using a Clarity^™^ Western ECL Substrate (Bio-Rad, CA, USA) and were visualized with an ImageQuant LAS 4000 imaging system (GE Healthcare, Little Chalfont, UK). Band intensities were quantified using ImageJ software (NIH, MD, USA) and normalized to β-actin.

**Wound healing assay and invasion assay**

For the wound healing assay, cells were seeded into a chamber of an SPL Scar™ Block (SPL Life Sciences, Pocheon, Korea) and cultured for 24 hours. After removing the blocks, cells were washed twice with DPBS and incubated in a medium for an additional 48 hours. The closed gap was quantified using ImageJ software. The invasion assay was performed with modifications to the protocol described by Jin et al. [3]. Briefly, Transwell inserts were coated with 100 μL of Matrigel (0.2 mg/mL; Corning, NY, USA) and incubated for two hours. Cells were seeded into the upper chambers in a serum-free medium, while the lower chambers contained a medium with 10% FBS. After 24 hours, non-invasive cells were removed, and invasive cells were stained with 1% crystal violet. Stained cells were counted in three randomly selected fields under a light microscope (Olympus CKX53).

**Cell proliferation assay**

Cells were seeded in 96-well plates and cultured for 48 hours. Cell proliferation was assessed using an EZ-Cytox assay kit (Daeil Lab, Seoul, Korea) according to the manufacturer’s instructions. Absorbance was measured with a UV-visible spectrophotometer, and the viability of the control group was set to 100% for normalization.

**Chemicals**

Gold (III) chloride trihydrate (HAuCl_4_) and branched poly-ethylenimine (PEI; Molecular weight (MW): 25,000) were purchased from Sigma Aldrich. Thiolated polyethylene glycol (PEG-SH; MW: 2,000) was obtained from SunBio (Anyang, Korea) and FA-modified PEG-SH (FA-PEG-SH; MW: 2,000) was purchased from Nanosoft Polymers (NC, USA).

**Preparation and functionalization of AuNPs**

The direct synthesis of AuNPs utilizing PEI by Song et al. was performed with slight modifications [4]. Briefly, a 1 mM HAuCl_4_ solution was refluxed at 100 ℃, to which a 3% PEI solution (MW: 25,000; Sigma Aldrich) was added. The mixture was cooled after five minutes, and the AuNP concentration was determined by UV-vis spectroscopy. For functionalization, AuNPs were first conjugated with a 1,000-fold molar excess of FA-PEG-SH for 15 hours. Subsequently, siESM (Bioneer; sense: 5′-CGUAUGCCACCUCAGAGAU-3′, antisense: 5′-ACUUCUGAGGUGGCAUACG-3′) was loaded onto the AuNP-FA by incubating with a 1,000-fold molar excess of siESM for one hour in the dark. After each step, unconjugated molecules were removed by centrifugation (16,100 × g, 15 min) and three washes. Unloaded siESM in the supernatant was analyzed to determine the loading ratio of AuNP-FA-siESM by means of a SYBR Gold assay [5]. The pellet was resuspended with nuclease-free water and washed three times by centrifugation. Finally, the synthesized AuNP-FA-siESM was characterized using transmission electron microscopy (Hitachi H-7100), a zeta potential analysis (Malvern Zetasizer 2000), and Fourier-transform infrared spectroscopy (Bruker Alpha Platinum ATR system).

**siRNA release kinetics**

The siRNA release from AuNP-FA-siESM was measured by incubating the particles in DPBS (pH 7.4) at 37 ℃. At predetermined time points, the supernatant was collected after centrifugation (16,100 × g, 15 min). The siRNA content in the supernatant was analyzed using a SYBR Gold assay, and the cumulative release was calculated as follows:

$$Cumulative siRNA release \left( \% \right)=(\frac{\sum\left( siRNA released at each timepoint \right)}{Initial siRNA loading})\times100$$

**Immunocytochemistry of AuNP-PEG or FA-siESM localization**

P2-MDA cells were seeded onto glass-bottom dishes. Transfection was performed with AuNP-PEG-Cy5.5-labeled siESM and AuNP-FA-Cy5.5-labeled siESM for 24 hours. Cells were washed, fixed with 4% paraformaldehyde, and stained with Alexa Fluor 488 Phalloidin (1:1,000, Invitrogen) and DAPI (Molecular Probes). Fluorescent signals were visualized using a confocal laser-scanning microscope (Zeiss LSM 880). Fluorescence intensity was analyzed in three regions of interest (ROIs) per sample and normalized to cell counts.

**Expression of adhesion molecules in HUVEC**

P2-MDA cells were treated with AuNP-FA or AuNP-FA-siRNA for 24 hours. A conditioned medium (CM) was collected by centrifugation at 450 × g for three minutes and filtered through a 40 μm cell strainer (MDA-CM). MDA-CM was diluted 1:1 with the HUVEC growth medium. HUVEC were treated with the respective MDA-CM specimens for 24 hours. Adhesion molecule expression was quantified using qRT-PCR and Western blotting. For immunocytochemistry, cells were fixed with 4% paraformaldehyde and stained with mouse anti-VCAM1 (Invitrogen) and mouse anti-ICAM1 (Abcam) primary antibodies. Fluorescent secondary antibodies conjugated to Alexa 594 goat anti-mouse (Invitrogen) and Alexa Fluor 488 Phalloidin (Invitrogen) and DAPI were applied for visualization.

**Statistical analyses**

Data were presented as the mean ± SD. Statistical significance was evaluated by means of one-way analysis of variance (ANOVA) with Tukey’s post hoc test for multiple comparisons or unpaired Student’s t-tests for two-group comparisons. Significance levels were set to ^*^*p* < 0.05, ^**^*p* < 0.01, and ^***^*p* < 0.001. GraphPad Prism was used for statistical analyses and data visualization.

**References**

1. Cai X, Luo J, Yang X, Deng H, Zhang J, Li S, et al. In vivo selection for spine-derived highly metastatic lung cancer cells is associated with increased migration, inflammation and decreased adhesion. Oncotarget. 2015;6(26):22905-17. http://doi.org/10.18632/oncotarget.4416.

2. Kim SJ, Ko W-K, Heo DN, Lee SJ, Lee D, Heo M, et al. Anti-neuroinflammatory gold nanocomplex loading ursodeoxycholic acid following spinal cord injury. Chem Eng J. 2019;375:122088. http://doi.org/https://doi.org/10.1016/j.cej.2019.122088.

3. Jin H, Rugira T, Ko YS, Park SW, Yun SP, Kim HJ. ESM-1 Overexpression is Involved in Increased Tumorigenesis of Radiotherapy-Resistant Breast Cancer Cells. Cancers (Basel). 2020;12(6). http://doi.org/10.3390/cancers12061363.

4. Song WJ, Du JZ, Sun TM, Zhang PZ, Wang J. Gold nanoparticles capped with polyethyleneimine for enhanced siRNA delivery. Small. 2010;6(2):239-46. http://doi.org/10.1002/smll.200901513.

5. Lobovkina T, Jacobson GB, Gonzalez-Gonzalez E, Hickerson RP, Leake D, Kaspar RL, et al. In vivo sustained release of siRNA from solid lipid nanoparticles. ACS Nano. 2011;5(12):9977-83. http://doi.org/10.1021/nn203745n.

**Supplementary Figure S1. a** Schematic illustration showing the synthesis of AuNP-FA-siESM via electrostatic interactions. **b** TEM images of AuNP, AuNP-FA, and AuNP-FA-siESM (left). Zeta-potential measurements of each AuNP formulation following sequential coating steps with FA-PEG and siESM (right). Results were analyzed by a one-way ANOVA with Tukey’s post hoc test (^***^*p* < 0.001). **c** FT-IR analysis of each AuNPs formulation, confirming stepwise modifications with PEI, PEG, and FA (left). Cumulative release kinetics of siRNA from AuNP-FA-siESM (right). **d** Confocal microscopy images of internalization of Cy5.5-labeled AuNP-PEG-siESM and AuNP-FA-siESM in P2-MDA cells (left). Quantitative analysis of Cy5.5 fluorescence intensities in P2-MDA cells (right). Results were analyzed by an unpaired Student’s t-test (^***^*p* < 0.001).


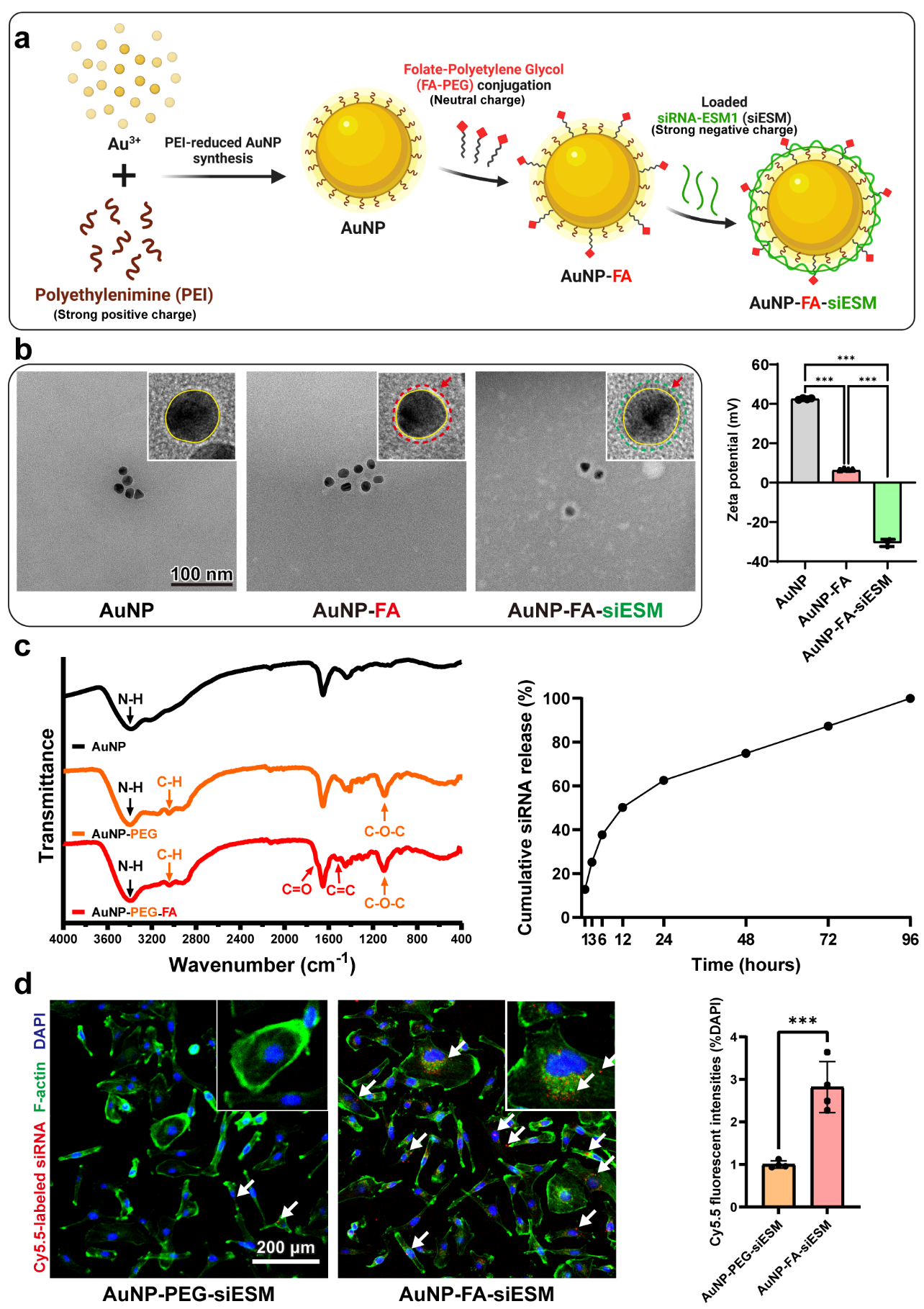


**Supplementary Table S1. Nucleotide sequences of primers used in real-time qRT-PCR.**

| Gene | Forward (5' → 3') | Reverse (5' → 3') |
| --- | --- | --- |
| ESM-1 | GCCCTTCCTTGGTAGGTAGC | TGTTTCCTATGCCCCAGAAC |
| VCAM-1 | CCGGATTGCTGCTCAGATTGGA | AGCGTGGAATTGGTCCCCTCA |
| ICAM-1 | GGCCTCAGTCAGTGTGA | AACCCCATTCAGCGTCA |
| P-Selectin | TCCGCTGCATTGACTCTGGACA | CTGAAACGCTCTCAAGGATGGAG |
| E-Selectin | TCAAGGGCAGTGGACACAGCAA | GGAAACTGCCAGAAGCACTAGG |
| GAPDH | GTCTCCTCTGACTTCAACAGCG | ACCACCCTGTTGCTGTAGCCAA |
